# Imaging in Autologous Breast Reconstruction

**DOI:** 10.3390/cancers16162851

**Published:** 2024-08-15

**Authors:** Janet C. Coleman-Belin, Joshua Barnett, Nima Khavanin, Jonas A. Nelson, Carrie S. Stern, Robert J. Allen

**Affiliations:** Division of Plastic and Reconstructive Surgery, Department of Surgery, Memorial Sloan Kettering Cancer Center, New York, NY 10065, USA; janet.coleman-belin@icahn.mssm.edu (J.C.C.-B.); khavann@mskcc.org (N.K.); nelsonj1@mskcc.org (J.A.N.); sternc2@mskcc.org (C.S.S.)

**Keywords:** breast neoplasms, breast reconstruction, autologous, microsurgery, deep inferior epigastric flap perforator, surgical flaps, ultrasonography, tomography, X-ray computed, magnetic resonance angiography, imaging techniques, artificial intelligence

## Abstract

**Simple Summary:**

As breast cancer rates rise, more patients choose breast reconstruction. Autologous breast reconstruction (ABR) may be more cost-effective and offer superior patient quality of life compared to prosthetic implant-based reconstruction but it involves heightened surgical complexity and extended operative time. Imaging facilitates surgical planning, patient selection, and reduces the time spent in the operating room, thereby enhancing the likelihood of surgical success and decreasing complication rates. Advanced imaging has also spurred innovative surgical techniques aimed at improving aesthetic outcomes and minimizing donor-site morbidity, including robotic-assisted deep inferior epigastric flap perforator (DIEP) flap harvest and stacked microsurgical flaps.

**Abstract:**

The evolution of imaging actively shapes clinical management in the field. Ultrasonography (US), computed tomography angiography (CTA), and magnetic resonance angiography (MRA) stand out as the most extensively researched imaging modalities for ABR. Ongoing advancements include “real-time” angiography and three-dimensional (3D) surface imaging, and future prospects incorporate augmented or virtual reality (AR/VR) and artificial intelligence (AI). These technologies may further enhance perioperative efficiency, reduce donor-site morbidity, and improve surgical outcomes in ABR.

## 1. Introduction

In the United States, breast cancer accounts for nearly one in three new cancer diagnoses in females each year, with the American Cancer Society estimating over 350,000 new invasive or in situ cases in 2023 alone [1]. For many of these women, breast reconstruction allows for a restoration of form and a subsequent improvement in self-confidence and psychosocial wellbeing [2]. In 2022, over 151,000 women opted to undergo breast reconstruction, and, while the majority were implant-based, over 22% elected for an autologous procedure [3]. Compared to implant-based reconstruction, autologous breast reconstruction (ABR) may be more cost-effective and improve patient quality of life [4].

For over 40 years now, the use of tissues from the lower abdomen has been described for breast reconstruction [5]. Although more recent advances in microsurgery and perforator dissections have opened the doors for several other potential donor sites, the abdomen has established itself as the preferred option with often ample donor tissues in the lower abdomen and a relatively inconspicuous scar without the need for changes in patient positioning [6,7,8]. In 2022, over 23,000 abdominally based reconstructions were performed, accounting for nearly 70% of autologous procedures [3].

Although a considerable amount of research has been dedicated to understanding the blood supply of abdominal flaps, specific details can vary significantly from one patient to another and potentially even impact the success of the procedure. Historically, surgeons had to rely on intraoperative dissection and experience to identify the number and location of perforators leading to inefficiency and increased operative times [9]. Without data on the branching patterns of the perforators throughout the abdominal tissues or their subfascial and intramuscular courses, decisions on which to include must be made blindly, which may increase the risk of abdominal morbidity and fat necrosis [9].

Preoperative imaging has been adopted to improve the safety and efficiency of surgery across a number of surgical disciplines. Within the realm of surgical oncology, preoperative localization of breast tumors allows for precision in the planning of a lumpectomy [10] whereas the imaging of head and neck or orthopedic tumors allows for a preoperative understanding of the stage of the cancer, the structures involved, the extent of the resection, and the required reconstruction [11,12,13]. It is therefore not surprising that reconstructive surgeons are able to apply the same methodologies to improve the delivery of care. Just as preoperative computer-aided design and manufacturing (CAD/CAM) can help to determine the optimal placement of dental implants at the time of a free fibula, preoperative vascular imaging of the abdomen allows the surgeon to determine the optimal combination of perforators for harvesting the abdominal tissues while minimizing injury to the underlying muscle [12,14,15].

Abdominally based ABR has undergone considerable evolution since the original transverse rectus abdominis myocutaneous (TRAM) flap described by Hartrampf et al. [5]. Harvesting one or more rectus abdominis muscles results in significant weakness of the abdominal wall with a high rate of postoperative bulges or hernias [15,16]. In 1992, the deep inferior epigastric artery perforator (DIEP) flap was introduced allowing for microsurgical transfer of the lower abdominal tissues in a muscle-sparing fashion [17]. This improvement in abdominal wall morbidity, however, comes at the cost of significant surgical complexity, as the dominant, often submillimeter perforators to the flap need to be identified on the abdominal wall and dissected through the fascia and muscle [15].

DIEP flaps, now considered the gold standard of abdominally based ABR [18], have opened the door for the use of several imaging technologies to aid in surgical planning for ABR. Proposed benefits of preoperative delineation of the vascular anatomy include the following:

1. Decreased operative time: Mapping the precise location, size, and course of each perforator streamlines flap design by allowing surgeons to know exactly which perforators to include before entering the operating room. This results in shorter operative times and decreased risk of complications associated with prolonged surgery.
2. Fewer complications: Flap perfusion is critical to successfully transferring a soft and supple flap without undue amounts of fat necrosis. Preoperative and intraoperative assessment of each perforator and their perfusion allows for the development of a plan that minimizes the risk of such complications.
3. Decreased donor-site morbidity: By delineating the subfascial and intramuscular courses of the perforators, surgeons can minimize the amount of healthy muscle and/or fascia that is sacrificed, thereby reducing donor-site morbidity.
4. Efficient patient selection: Preoperative imaging can help surgeons assess the suitability of patients for abdominal-based free flaps, particularly if they have undergone previous abdominal surgery that may have threatened the flap’s perforators and/or pedicle.

To this end, several technologies have been described to improve the preoperative evaluation and intraoperative assessment of patients undergoing autologous breast reconstruction. This review aims to synthesize the state of the art in this highly active area of research, mapping out its evolution and delineating the downsides and utility of the various imaging techniques currently available for use. More specifically, we aim to understand the mechanism by which technologies including ultrasonography (US), computed tomography angiography (CTA), magnetic resonance angiography (MRA), fluorescent indocyanine green (ICG) angiography, and more novel three-dimensional (3D) surface imaging and virtual or augmented reality (VR/AR) can be applied in the planning of autologous breast reconstruction in order to improve surgical precision, outcomes, and patient satisfaction.

## 2. Ultrasonography (US)

The earliest attempts at such preoperative planning relied on handheld Dopplers and/or ultrasonography (US) alone. By primarily allowing surgeons to localize perforators on the abdominal wall, the anatomic data provided by these studies promised to minimize the time spent delineating the anatomy intraoperatively in a relatively low-cost fashion [19]. Color duplex ultrasonography provided the added benefit of visualizing flow through each perforator in real time; however, its resolution fell short of truly delineating their subcutaneous plexuses and the quality of images can be limited by patient anatomy and operator experience [20].

Ultrasound is a powerful, non-ionizing tool that can elucidate circulatory patterns within tissue in autologous breast reconstruction. The use of ultrasound has evolved significantly since the first commercial ultrasound became available in 1963 [21]. Sixty years later, ultrasound machines have advanced from large bulky machines to devices comparable to the size of cell phones [22]. In 1990, Dr. Taylor first used the now ubiquitous acoustic Doppler probe to pinpoint skin perforators to be used to ensure the viability of random pattern flaps [19]. This concept has been expanded upon and other ultrasound modalities are now used in plastic surgery, such as color duplex ultrasound and laser Doppler flowmetry. The compact and portable Doppler, which can be found in nearly every operating room, identifies moving objects relative to itself [23]. An acoustic Doppler can identify intravascular blood flow, differentiate between high-flow arterial blood and low-flow venous blood, and monitor flap perfusion postoperatively [24].

Innovations to Doppler and US technology include color and frequency. A color spectrum can be added to the moving component of an ultrasound to allow for color duplex imaging [25]. The observed color depends on the direction of flow relative to the transducer and can be manually selected. Typically, arteries are assigned red and veins are assigned blue. Newer transducers that allow for high-frequency monitoring are capable of scanning at more superficial depths and can identify vessels less than 0.5mm in diameter [26]. By utilizing high-frequency color duplex imaging, perforator take-off can be identified and the perforator can be characterized as intramuscular, submuscular, or suprafascial [27]. High-frequency imaging can be useful in identifying the perforator anatomy of a number of flaps used for breast reconstruction including TRAM and DIEP flaps.

Although not used for preoperative surgical planning, laser Doppler flowmetry has proven to be a powerful tool for postoperative flap monitoring. Laser Doppler flowmetry relies on tissue illumination by coherent laser light. The Doppler shift of the reflected laser can allow for the detection of blood flow within a given flap [28]. Real-time measurements of blood flow to a flap can be displayed remotely so that surgical team members can monitor the flap from afar. Although a powerful method, false positives can be observed even when there is no perfusion to the flap due to vibration of the tissue or patient movement [29]. Intraoperative laser Doppler flowmetry can be used to compare relative perfusion zones of the abdomen, especially after microsurgical anastomosis or during inset of the flap. False negatives can be found if the fiberoptic cable becomes dislodged from the flap or if a clot interferes with the interface [30].

Ultrasound was the first imaging modality widely accepted for ABR and has undergone advancements that improve its use in autologous reconstruction. Newer technologies, such as CTA and MRA, are now available and may offer superior perforator imaging.

## 3. Computed Tomography Angiography (CTA)

CTA provides a fast and simple alternative that more accurately delineates perforator characteristics, but without the ability to dynamically evaluate flow and at the cost of radiation and iodinated contrast exposure [31]. Combining the principles of traditional computed tomography (CT) with contrast-enhanced imaging to visualize blood vessels and assess blood flow, CTA has evolved as a crucial diagnostic imaging technique. The roots of CTA can be traced back to the development of CT itself in the early 1970s [32]. The invention of the CT scanner by Sir Godfrey Hounsfield and Dr. Allan Cormack revolutionized medical imaging by providing detailed cross-sectional images of the body [33]. However, early CT scans primarily focused on anatomical structures, and the visualization of blood vessels was limited.

The significant advancement toward CTA occurred in the 1980s when the introduction of intravascular contrast materials allowed for the visualization of vascular structures with greater clarity [33]. This innovation marked the inception of contrast-enhanced CT angiography. Over the years, technological improvements, such as faster scanning times, increased spatial resolution, and more sophisticated contrast agents, have continually refined CTA.

**Figure 1:**
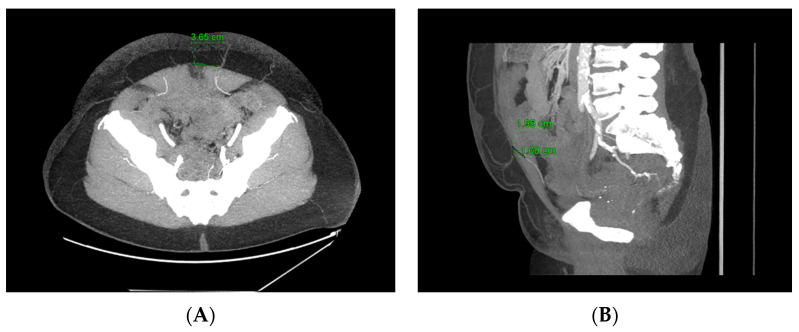
Computed tomography angiography (CTA) scan indicating a good candidate for a robotic-assisted deep inferior epigastric perforator (DIEP) flap due to the single dominant perforating vessel’s short intramuscular course (<3 cm long). The left deep inferior epigastric artery (DIEA) has a type 1 branching pattern. The perforator penetrates the fascia 6.0 cm below umbilicus reference point and 3.7 cm to the left (**A**). The subcutaneous segment courses anteriorly and branches throughout the subcutaneous fat. The subfascial segment is 1.1 cm long, coursing caudal (**B**). The intramuscular segment is 2.0 cm long, coursing caudal and lateral deep to muscle to join the main trunk, which courses 7.4 cm caudally.

CTA plays a significant role in the context of breast reconstruction, particularly in the preoperative planning and assessment of autologous-based reconstruction. CTA provides detailed imaging of the vascular anatomy and allows for precise identification of perforators within a given flap (Figure 1). CTA can help elucidate the unpredictable location of perforators, the number and size of perforators, and the intramuscular trajectory of these vessels. The preoperative planning of these perforators may allow surgeons to select the most reliable perforators, decrease operative time, and contribute to improved surgical outcomes [34].

Of the various imaging modalities, CTA has proven itself to be the most commonly preferred study. It is a fast and prevalent modality that allows for the accurate demonstration of the location, caliber, and course of perforators. CTA has been shown to be more accurate than ultrasound and more cost-effective than MRA [35,36]. CTA allows for volume-rendered reformats to visualize the subcutaneous, subfascial, and intramuscular pathways of perforators that can be used to perfuse abdominally based free flaps for breast reconstruction [37]. These reformats are overlaid with a grid to demonstrate the location of perforators in relation to surface landmarks such as the umbilicus. This allows for precise marking of the patient preoperatively. Radiological reports include the caliber of the perforators, the location of the perforators from the umbilicus and midline, and the length of the intramuscular course [37].

The most notable disadvantage of CTA is radiation exposure to the patient, particularly in populations at high risk for developing cancer [38]. A typical CTA of the abdomen and pelvis is associated with radiation doses of 10mSv, whereas a full trauma CT is associated with radiation doses of 25mSv. To give these values context, a CT of the abdomen and pelvis (CTAP) for preoperative surgical planning is the equivalent of 4 years of background equivalent radiation. Despite radiation exposure, benefits to the patient of CTA are considerable, including operating time and postoperative outcomes such as decreased flap loss and fat necrosis [33].

It is important to note that, when already available, previously obtained conventional CT or CT/PET scans of the abdomen and pelvis are acceptable alternatives to CTA in preoperative planning, sparing the patient radiation associated with a repeat scan. Recent comparisons of conventional CT and CTA found greater than 90% reliability for conventional CT in identifying the largest perforators, delineating intramuscular course, and demonstrating communications with and evaluating the superficial system [39,40].

Compared to ultrasound, CTA more clearly visualizes perforators at the cost of radiation exposure to the patient. Unlike MRA, neither ultrasound nor CTA offers quality imaging of surrounding soft tissue.

## 4. Magnetic Resonance Angiography (MRA)

MRA promises a radiation-free alternative with the potential for excellent soft tissue resolution, though imaging can be time-intensive and claustrophobic for patients [41].

Since the first proof of concept was published in 1985, magnetic resonance angiography (MRA) has become an important modality in the evaluations of vasculature across the body, including the neck, brain, and even the coronary arteries [42]. Some of the earliest reports in applying the technology to abdominally based breast reconstruction were published in 2009 [43,44]. In contrast to CTA, MRA utilizes radio waves to measure the activity/relaxation time of hydrogen nuclei under the effect of a strong magnetic field. Multiple radiofrequency pulses can be used in sequence to emphasize particular tissues, thereby delineating tissues with different inherent relaxation times [45].

**Figure 2:**
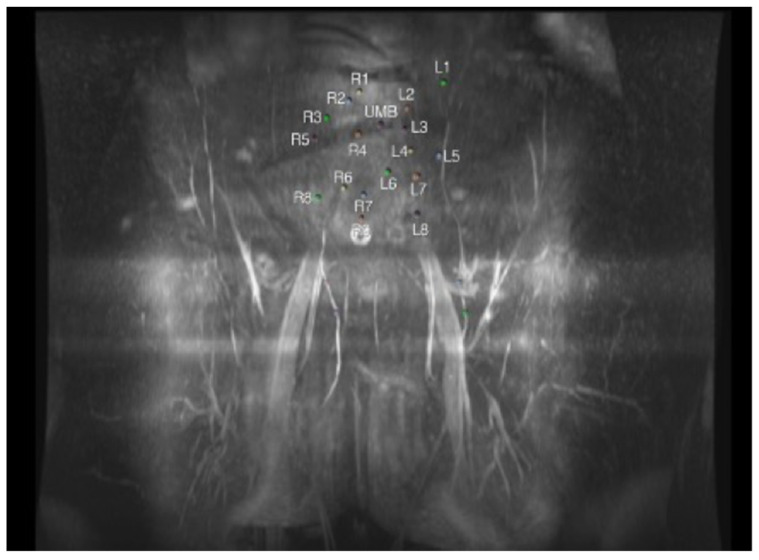
Magnetic resonance angiography (MRA) perforator mapping of the abdomen. The image shows patient right (R) and left (L) perforator locations in relation to the umbilicus (UMB).

One of the earliest studies on MRA in preoperative planning for DIEP flaps found a decreased accuracy in identifying perforators, which the authors attributed to MRA’s low spatial resolution [44]. Since then, however, there have been considerable advances and fine tuning to MRA protocols, and many consider the image quality to be equivalent, if not superior, to that of CTA, with excellent muscle-to-vessel-contrast resolution (Figure 2) [46]. In fact, the resolution is so great that more recently MRA has been used by researchers to evaluate the relationship and anastomoses between the superficial and deep inferior epigastric vasculature within the subcutaneous fat [47]. Recent comparisons of MRA to CTA have demonstrated an equivalent, if not improved, ability to delineate the vascular anatomy [35,41,48].

The primary advantage of MRA is that it does not typically expose patients to harmful ionizing radiation [44,45,49]. This is particularly important in patients with an underlying predilection for cancer formation, including those with a breast cancer 1 or 2 (BRCA1/2) or tumor protein 53 (tp53) mutation.

The primary disadvantages of MRA resolve around its relatively expensive cost, potentially decreased access/prolonged wait times, and more time-consuming studies. It is not uncommon for patients to require pharmacologic anxiolysis prior to the study due to claustrophobia [49]. Furthermore, although MRA avoids the iodine-based contrast agents used in CTA, the use of non-iodinated contrast agents such as gadolinium comes with its own set of risks. Although generally considered less nephrotoxic, gadolinium was recently the subject of an FDA warning for its tendency to deposit in patients’ bodies, particularly in the brain of patients with underlying chronic kidney disease [49]. In order to avoid contrast agents, perforator phase contrast angiography (pPCA) has been suggested as a non-contrast MRA technique that offers superior image quality to CTA [50]. Finally, the use of MRA is generally contraindicated in patients with metal-containing implants, which, at some centers, may include breast tissue expanders.

**Figure 3:**
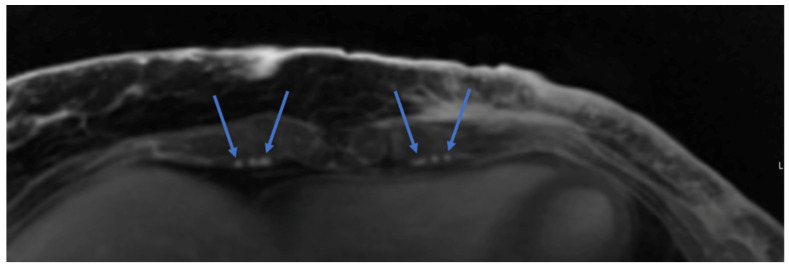
Magnetic resonance angiography (MRA) of the chest for preoperative planning. The blue arrows indicate internal mammary veins on each side of the artery bilaterally.

MRA is commonly employed in our practice and allows for detailed preoperative assessment of the pedicle course, perforator anatomy, and subcutaneous perforator angiosome. The size, course, and branching patterns of the deep inferior epigastric artery (DIEA) and vein (DIEV) as well as the superficial system are clearly delineated. The deep circumflex iliac system is also clearly visualized as it branches off the external iliac arteries and provides an excellent secondary flap option when the deep inferior epigastric system is not viable [41]. MRA including the chest can also evaluate the internal mammary arteries and veins as potential recipients for microsurgical anastomosis (Figure 3) [41]. Where MRA shines is in its excellent ability to demonstrate the intramuscular course of perforators. This detailed information allows for a much safer and simpler intramuscular dissection, speeding up the flap harvest and allowing surgeons to understand exactly how much intervening muscle needs to be sacrificed in order to include two or more nearby perforators within a flap.

**Figure 4:**
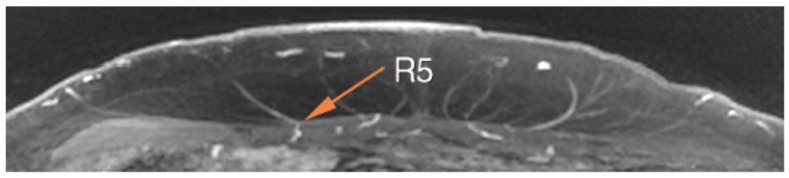
Magnetic resonance angiography (MRA) of the abdomen with perforator mapping. R5 is located 9.0 mm inferior to the umbilicus and 44.3 mm to the right of midline. The perforator can be seen coursing laterally though the subcutaneous tissues, primarily perfusing the skin and fat of Hartrampf zone III. The vessel diameter is 1.5 mm. The perforator drops straight down through the fascia to meet with the lateral intramuscular branch of the deep inferior epigastric artery (DIEA).

**Figure 5:**
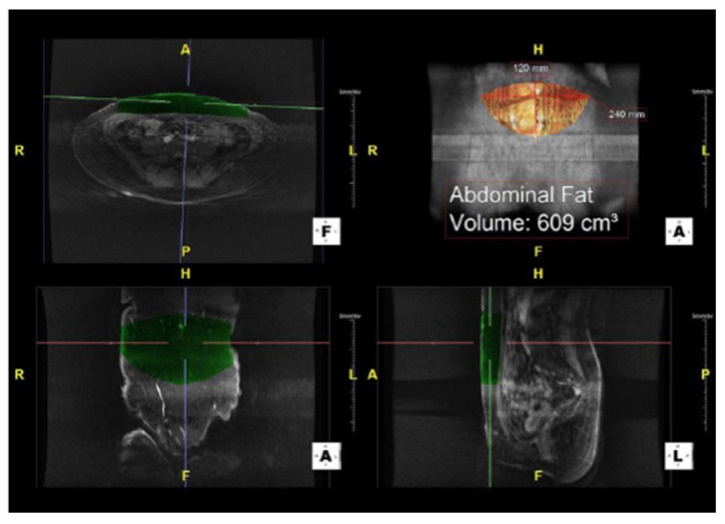
Magnetic resonance angiography (MRA) image with volumetric analysis of the abdominal donor site. Anatomical locations and views are labeled: anterior (A), posterior (P), right (R), left (L), head (H), and feet (F).

Finally, MRA technology allows for a clear demonstration of not only a perforator’s size, but also the branching pattern of its course throughout the subcutaneous fat (Figure 4). This allows surgeons to ensure adequate flap perfusion while preoperatively planning which perforators to include. Combined with computer estimates of abdominal tissue volume, surgeons can also come up with more objective and accurate estimates of flap volume (Figure 5). Particularly in large-breasted women, this can facilitate decisions regarding the need for stacked flaps or hybrid (implant and autologous) reconstructions [51,52,53].

CTA or MRA images can be projected onto the patient’s flap donor site to intraoperatively visualize the patient’s vascular anatomy [54]. While frequently utilized in neurosurgery, these models are uncommon in plastic and reconstructive surgery (PRS) and serve the basis of augmented and virtual reality (AR/VR) applications to be discussed later [55]. Other emerging technologies include projections built by 3D software based on a patient’s two-dimensional (2D) CTA or MRA images (Figure 2); surgeons can then highlight the most favorable perforator, project the 3D virtual model onto the patient using the umbilicus as a landmark, mark perforator locations, and confirm guidelines with unidirectional Doppler US. These projections may improve on the limitations of the unidirectional Doppler, including the inability to differentiate desired arterial vessels from fascial perforator arteries and to assess arterial branching patterns [54].

In many ways, MRA incorporates the best of both ultrasound and CTA. Like ultrasound, MRA does not expose the patient to radiation, and like CTA, MRA clearly visualizes perforators. MRA also offers superior soft tissue imaging when compared with both other modalities. However, these benefits are time-consuming; while ultrasound or CTA can be completed in a few minutes, MRA imaging can take over an hour and may be claustrophobic for some patients. Although objective data on current usage trends for each technology are lacking within the literature, the availability and ease of CTA has established it as the preferred option for many autologous breast reconstruction practices worldwide. MRA and high-frequency ultrasound are gaining traction as attractive, radiation-free alternatives; however, their widespread utilization is limited by availability and cost [49,56].

## 5. Dye-Based and Indocyanine Green (ICG) Angiography

**Figure 6:**
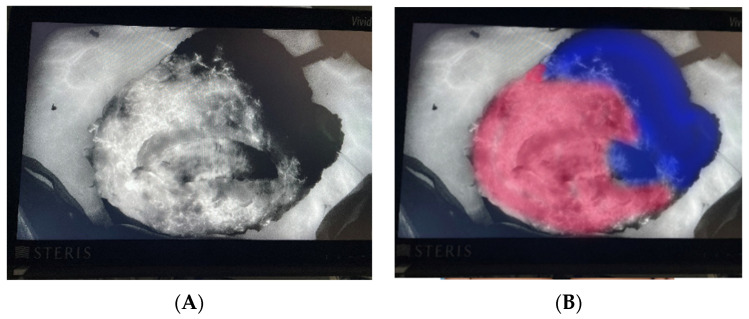
Indocyanine green (ICG) angiography to evaluate intraoperative perfusion of a deep inferior epigastric perforator (DIEP) flap. (**A**) Intravenously injected ICG dye appears as a bright gray/white color in tissues when evaluating with the SPY portable handheld imager (SPY-PHI) thereby demonstrating active perfusion to those tissues. Areas of the flap appearing dark black do not exhibit ICG and are therefore not perfused (or are under-perfused). (**B**) The unlit area (overlaid in blue) is poorly perfused and must be trimmed intraoperatively. The brightest tissue areas (overlaid in red) demonstrate the best perfusion.

While CTA, MRA, and US have dominated ABR imaging modalities, innovations in imaging intraoperatively may improve surgical and patient outcomes. Fluorescent imaging technology using dye-based angiography has become particularly prevalent in microsurgical ABR. The SPY (SPY Elite; Stryker, Kalamazoo, MI) was developed in 2005 and adopted for use in plastic surgery in 2011 in order to assess blood flow and flap perfusion intraoperatively [57]. Once isolated on the perforators or following completion of the microsurgical anastomoses, the anesthesiologist injects intravenous dye, usually indocyanine green (ICG), to be visualized on a computer screen using a laser-assisted portable handheld imager (PHI). Perfused tissue is brightly lit on-screen due to fluorescent dye; malperfused tissue, however, remains dark (Figure 6) [58]. Therefore, SPY-PHI ICG angiography can assess overall anastomotic success, as well as any focal areas of the flap that lack blood flow. These non-perfused segments can then be trimmed by surgeons intraoperatively. In autologous breast reconstruction, SPY-PHI use has been associated with significantly lower odds of fat necrosis and partial flap loss [59].

Fluorescent imaging can also be used to assist in perforator selection and the assessment of mastectomy skin flap perfusion [59,60,61,62]. By selective clamping of perforators, the surgeon can use this imaging to determine the need for the inclusion or exclusion of additional perforators. This not only ensures adequate perfusion of the flap but can limit morbidity by excluding perforators that are not essential to the blood supply of the flap [60,61,62]. Being able to assess the mastectomy skin flap perfusion at the same time for patients undergoing immediate reconstruction is another added benefit of using ICG angiography intraoperatively [60]. Poorly perfused mastectomy skin can be selectively debrided and replaced with flap skin. Mastectomy skin flap necrosis can result in prolonged wound care, the need for additional surgeries, abnormal scarring, and decreased patient satisfaction [63,64]. Overall, ICG angiography has become a powerful tool for the microsurgeon as a way to assess both flap and mastectomy skin perfusion and the selection of perforators intraoperatively.

## 6. 3D Surface Imaging

MRA or CTA can estimate the volume of soft tissue available for breast flap reconstruction based on standard measurements; however, these volumes are often inaccurate in the operating room [65]. As a result, 3D surface imaging has arisen as a possible imaging tool for ABR with applications in preoperative planning, volumetric analysis, and patient education [66,67]. 3D surface imaging software is gaining popularity as an adjunct technology to assist in flap inset, allowing for improved breast projection and overall cosmesis [68]. Commercially available examples include VECTRA (Canfield Scientific, Parsippany, NJ, USA), Go!Scan 3D Scanner (Creaform, Levis, QC, Canada), Crisalix (Crisalix SA, Lausanne, Switzerland), or the Vivid 900 or 9i 3D Digitizer (Konica Minolta Inc., Tokyo, Japan). It is not uncommon for modern practices to employ one or more of these techniques in combination, using their complimentary data to maximize patient outcomes.

Surgical teams can use 3D surface imaging software to address patient expectations and discuss revision options to jointly create the best overall reconstruction. VECTRA, considered the 3D imaging modality of choice in plastic surgery, originated in cosmetic surgery in 2009 and was later applied to reconstructive ABR [69]. This system swiftly captures photos of patients in clinic and allows surgeons to simulate morphologic changes, analyze breast asymmetry, and conduct volumetric analysis. Using longitudinal stereophotogrammetric 3D photos, patients and surgeons can use 3D imaging data to discuss the objective size and shape of the reconstructed breast(s) in clinic along with areas that feel volume-deficient [66]. These photos can be paired with surgical algorithms in order to focus on the areas of highest need that the human eye may miss, such as volume-deficient areas in need of fat grafting. Additionally, 3D imaging can simulate the impact of a procedure before the intervention. Postoperative follow-up advantages include accurate tracking of objective results over time, surpassing the limitations of subjective 2D images [66,67]. 3D surface imaging can also aid 3D printing to create precise breast molds. Especially in unilateral reconstructions, these 3D printed molds may be useful for shaping thin and flexible flaps into a shape symmetric to the contralateral breast [70].

3D surface imaging software is highly promising for the future of imaging in ABR, including its potential applications to AR/VR technology [66,68]. The most notable drawback is a lack of research correlating objective 3D surface imaging with a patient’s physical exam. Additionally, 3D surface imaging is currently unable to identify accurate depth [66]. The lack of objective skin envelope thickness may lead to discrepancies between estimated and real tissue volume needed for ABR [66]. Volume predictions are further limited because the software estimates the location of the rectus sheath. As a result, cross-sectional CTA and MRA images are still needed to calculate accurate flap depth.

## 7. Current State-of-the-Art and Future Clinical Applications

Access to high-quality imaging in ABR has fostered innovative surgical techniques, including “stacked” flaps and robotic-assisted DIEP flaps, and provided the baseline for future clinical applications like virtual/augmented reality and artificial intelligence.

### 7.1. Imaging in Stacked Flap Breast Reconstruction

For some patients seeking autologous breast reconstruction, a single hemiabdomen does not provide enough volume for the reconstruction. In this scenario, additional volume can be added by “stacking” flaps from the abdomen or alternative donor sites (i.e., thighs, lower back, or buttock) [18,71,72]. While the internal mammary vessels are the workhorse recipient vessel in ABR, the inclusion of another flap calls for an additional set of recipient vessels for the second flap. There are several options to consider, as follows: thoracodorsal vessels, going retrograde to the internal mammary vessels, or “daisy chaining” of the flaps [18,71,72]. While each option is reasonable, preoperative imaging allows for their evaluation ahead of time. Abdominal imaging can identify patients that have ideal branching patterns for daisy chaining of the flaps [18]. Ideal candidates for this scenario have a large superior runoff of the deep system or a type 2 system with a large branch coming off the main deep inferior epigastric pedicle. As a preference, we frequently go antegrade and retrograde to the internal mammary vessels when stacking flaps. Preoperative imaging of both the donor and recipient site helps us better plan the procedure and counsel our patients.

Volumetric analysis of donor sites also allows for the estimation of donor-site volume and the identification of patients that are likely to require additional volume beyond a single hemi- or extended hemi-abdominal flap for ABR. These data can be used to counsel the patient on the anticipated length of surgery, the need for additional donor sites, and potential added recovery postoperatively [73].

### 7.2. Recipient Vessel Assessment

As we alluded to earlier, in our practice, we rely on imaging to evaluate not only the abdominal tissues, but the recipient vessels as well. In particular, in patients with a history of axillary lymph node dissection and/or adjuvant radiation, preoperative MRA or CTA clearly delineates the vascular anatomy of the internal mammary and thoracodorsal systems [74]. Knowing the branching pattern of the internal mammary vein is helpful in determining which intercostal space to approach, as veins less than 2 mm in diameter may not provide sufficient outflow for the flap or may present a size match discrepancy that can result in venous thrombosis [18]. In patients with favorable internal mammary perforators, one can even plan to avoid dissection of the main artery and vein and perform the anastomosis to the perforating vessels [75]. Additionally, imaging allows for the evaluation of the thoracodorsal system for injury and surrounding scar tissue.

### 7.3. Robotic-Assisted DIEP Flap Harvest

Robotic-assisted DIEP flaps are gaining popularity as an approach to minimize donor-site morbidity and maximize pedicle length in ABR [76,77,78,79,80]. Not all patients are candidates for robotic-assisted DIEP flaps, however. A CTA or MRA of the lower abdomen is essential for identifying which patients who are candidates (Figure 1). Patients are ideal candidates when imaging indicates a significant perforator within the flap and with a short intramuscular course (<3–4 cm) [76,80]. Kurlander et al. evaluated 49 preoperative CTAs and found that 71% of hemiabdomens appropriate for robotic harvest [80]. Robotic harvest limits the disruption of the anterior rectus sheath, minimizes division of the rectus abdominus muscle, and spares motor nerves [76,77,78,79]. To this effect, the robotic DIEP flap may limit patient morbidity and improve outcomes in breast reconstruction [17,76,77,78,79,80]. This latest evolution in ABR would not be possible without the use of preoperative imaging.

## 8. Augmented Reality (AR), Virtual Reality (VR), and Artificial Intelligence (AI)

Future directions include applying augmented reality (AR), virtual reality (VR), and artificial intelligence (AI) to breast reconstruction to further improve the surgical experience for patients, surgeons, and surgical trainees.

Although not widespread, the use of AR/VR to visualize the anatomy and pertinent operative steps has significant promise in ABR. Proposed benefits include expeditious, accurate, and non-invasive AR-assisted vessel identification. A feasibility study by Fitoussi et al. (2021) reports a median distance of 2 mm between AR and Doppler landmarks, demonstrating the potential of AR in precise preoperative mapping for DIEP flap surgery [81]. AR/VR glasses and headsets allow for an immersive preoperative 3D model of the patient’s anatomy and can facilitate quicker and more precise dissection of perforators intraoperatively [82]. Additionally, both AR/VR are promising educational tools to train residents and medical students [13,83,84]. Limitations include the time-consuming creation of 3D AR models and the need for further validation for intraoperative use [83].

Integrating artificial intelligence (AI) with imaging may be the next frontier for assisting ABR surgical planning. AI can be combined with any of the previously mentioned 2D or 3D imaging techniques, as well as with AR/VR, for automated vessel identification, perforator mapping, and flap viability prediction [84,85].

## 9. Conclusions

More patients have opted for breast reconstruction as the incidence of breast cancer increases [3]. ABR may be more economical and improve patient quality of life compared to prosthetic implant-based reconstruction [4], but it comes at the cost of increased surgical complexity and prolonged operative time [15]. By aiding surgical planning, improving patient selection, and decreasing the time patients spend in the operating room under anesthesia, imaging may increase the likelihood of surgical success and decrease complication rates [15]. High-quality imaging has additionally fostered innovation aiming to further decrease donor-site morbidity, including robotic-assisted DIEP flap harvest and stacked flaps [76,86]. Ultimately, imaging has dramatically changed clinical management in ABR.

Computed tomography angiography (CTA), ultrasound (US), and magnetic resonance angiography (MRA) are the most prominently researched ABR imaging modalities. Emerging technologies, including “real-time” ICG angiography imaging and three-dimensional (3D) surface imaging, and future directions incorporating augmented or virtual reality (AR/VR) and artificial intelligence (AI), may offer novel insights to further improve perioperative efficiency, reduce donor-site morbidity, and enhance cosmetic and surgical outcomes. See Table 1 for an overview of the utility and limitations of each imaging modality.

This review serves as a summary of the various imaging methods currently used for preoperative planning and operative decision making in autologous breast reconstruction. As such, this review is limited by the available studies within this clinical scope.

Because each imaging modality has unique strengths and limitations, the next steps for assessing the utility of each include long-term objective and patient-reported outcomes. Current measures of outcomes in ABR are primarily in patient-reported outcome measures (PROMs) such as the BREAST-Q [87]. However, these quality-of-life outcomes have yet to be robustly compared between the presence or lack of imaging and between the imaging modalities themselves. Further prospective studies are needed to evaluate the longitudinal impact of image-guided ABR techniques.

## Figures and Tables

**Table 1:** Summary of imaging modalities used in autologous breast reconstruction.

Imaging Modality	Perforator Location	Perforator Size	Intramuscular Anatomy	Subcutaneous Branching	Superficial System	Recipient Vessels	Flow Assessment	Perfusion Assessment	Downsides
Ultrasound	Yes	No	No	No	No	No	Yes	No	Operator dependent
CTA	Yes	Yes	Variable	Variable	Yes	Yes	No	No	Ionizing radiation
MRA	Yes	Yes	Yes	Yes	Yes	Yes	No	No	Costly
Dye-based angiography	Yes	No	No	No	No	No	No	Yes	Limited to surface
3D surface imaging	NA	NA	NA	NA	NA	NA	NA	NA	Software limitations

## Data Availability

The original contributions presented in the study are included in the article, further inquiries can be directed to the corresponding author/s.
